# Cesarean scar ectopic pregnancy: invasion of the bladder wall detected by magnetic resonance imaging

**DOI:** 10.1590/0100-3984.2014.1855

**Published:** 2017

**Authors:** Nelson Marcio Gomes Caserta, Angela Maria Bacha, Oswaldo R. Grassiotto

**Affiliations:** 1 PhD, Tenured Associate Professor, Department of Radiology, Faculty of Medical Sciences, FCM-Unicamp, Campinas, SP, Brazil.; 2 PhD, Professor, Department of Gynecology and Obstetrics, Faculty of Medical Sciences, FCM-Unicamp, Campinas, SP, Brazil.

**Keywords:** Pregnancy, ectopic, Cesarean section, Urinary bladder, Hematuria, Magnetic resonance imaging

## Abstract

Although cesarean scar ectopic pregnancy continues to be the rarest form of ectopic pregnancy, its incidence is increasing because of the worldwide increase in the number of cesarean deliveries. If the diagnosis is delayed, there is a high risk of severe hemorrhage and death, whereas early diagnosis can minimize the complications associated with the condition. Here, we report a case in which invasion of the bladder wall was identified by magnetic resonance imaging.

## Abstract (pt)

Gestação em cicatriz de cesariana é a forma mais rara de gravidez ectópica, mas com aumento devido ao maior número de cesarianas. O diagnóstico tardio pode provocar hemorragia grave, com risco de morte, mas se precoce pode reduzir as complicações. Relatamos um caso com invasão da parede da bexiga, provocando hematúria, e que foi demonstrada pela ressonância magnética.

## INTRODUCTION

Cesarean scar ectopic pregnancy is a rare form of ectopic pregnancy that is considered a potentially life-threatening condition^(1,2)^. Invasion of the myometrium may lead to massive uterine bleeding^(3)^. We report a case of cesarean scar ectopic pregnancy that invaded the bladder wall, which was confirmed by magnetic resonance imaging (MRI).

## CASE REPORT

**Figure 1:**
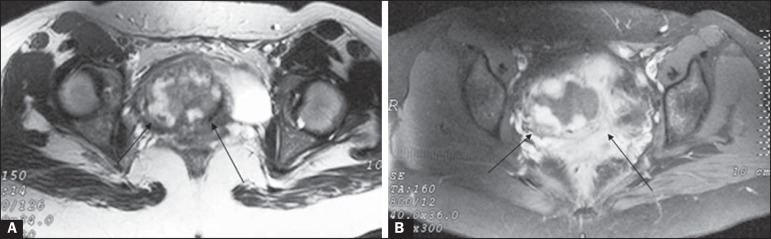
**A:** Axial T2-weighted MRI showing a heterogeneous mass on the right side of the uterine isthmus (arrows). **B:** After administration of gadolinium, there was pronounced, heterogeneous impregnation of this mass (arrows).

**Figure 2:**
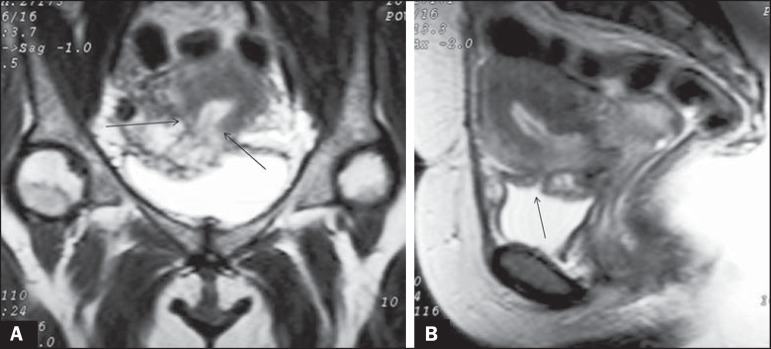
**A:** Coronal T2-weighted MRI showing that the myometrium (arrows) was ruptured by the gestational mass. **B:** Sagittal T2-weighted MRI scan along the midline, showing the empty endometrial cavity and the opening (arrow) caused by the cesarean scar ectopic pregnancy invading the bladder.

A 37-year-old patient (gravida 5, para 4) was referred to our hospital with a three-week history of macroscopic hematuria and painless vaginal bleeding. On the basis of the clinical evaluation, it was determined that she was again pregnant, and the gestational age was estimated to be at least ten weeks. The patient was hemodynamically stable and had undergone four cesarean sections without complications. A transabdominal ultrasound showed a heterogeneous anembryonic mass in the lower uterine segment, with hypervascularization and apparent extension to the bladder wall. The serum level of hCG was 38.8 mIU/mL (the expected range for a normal pregnancy at 10 weeks is 25,700-288,000 mIU/mL). MRI revealed a heterogeneous hyperintense mass of the myometrium in the lower anterior uterine segment (Figure 1). At one point, the mass had invaded the bladder wall and opened through an orifice in the bladder mucosa, as identified on the MRI scan (Figure 2). Cystoscopy confirmed the opening of the fistula, and a biopsy of this site revealed chronic cystitis. The patient was submitted to hysterectomy with resection of the bladder wall lesion. Anatomopathological examination confirmed the diagnosis of ectopic pregnancy in a cesarean scar with invasion of the bladder wall. She developed no complications during the postoperative period.

## DISCUSSION

Implantation of a pregnancy within the scar of a previous cesarean section is a potentially life-threatening condition and is considered the rarest form of ectopic pregnancy^(4)^. It is known that cesarean section represents one of the risk factors for ectopic pregnancy and placental abnormalities in subsequent pregnancies^(5)^. Although many hypotheses have been proposed for this rare condition, the most reasonable explanation would be that the trophoblast penetrates the myometrium along a microscopic tract^(6)^.

Early diagnosis with ultrasound can offer treatment options that could prevent uterine rupture and hemorrhage and thus preserve the uterus^(4)^. Curettage seems contraindicated because the trophoblastic tissue is outside the uterine cavity^(4)^. Nonsurgical treatment options include administration of systemic and local methotrexate, as well as potassium chloride and hyperosmolar glucose, which have reportedly met with some success ^(4,5,7)^. However, primary surgical treatment by laparotomy and hysterotomy, as soon as the diagnosis is confirmed, would be the best treatment option^(4)^.

Clinical history and endovaginal ultrasound are quite useful for differentiating cesarean scar ectopic pregnancy from incomplete abortion or cervico-isthmic gestation. Our patient presented with macroscopic hematuria, which is not expected as a symptom of cesarean scar ectopic pregnancy. Approximately 40% of patients with cesarean scar ectopic pregnancy experience only painless vaginal bleeding^(1)^.

Some authors have used MRI as an additional diagnostic modality. A recent report indicated that contrast-enhanced MRI can be used as the initial imaging modality to diagnose cesarean scar ectopic pregnancy, in selected cases, allowing a more accurate diagnosis before the specific treatment is instituted^(8)^. Because MRI has excellent tissue resolution, it can be used in order to locate the implantation in the cesarean section scar, determine the thickness of the anterior uterine wall, and provide an accurate view of the vesicouterine space. Although invasion of the bladder wall is a known possibility in cesarean scar ectopic pregnancy, we know of no other reports of this complication diagnosed by MRI. In the case presented here, MRI clearly demonstrated that the hematuria was caused by the penetration of the ectopic pregnancy into the bladder wall.
